# DNA-immunisation with dengue virus E protein domains I/II, but not domain III, enhances Zika, West Nile and Yellow Fever virus infection

**DOI:** 10.1371/journal.pone.0181734

**Published:** 2017-07-25

**Authors:** Jose L. Slon Campos, Monica Poggianella, Sara Marchese, Monica Mossenta, Jyoti Rana, Francesca Arnoldi, Marco Bestagno, Oscar R. Burrone

**Affiliations:** 1 Molecular Immunology Group, International Centre for Genetic Engineering and Biotechnology, Trieste, Italy; 2 Department of Medicine, Surgery and Health Sciences, University of Trieste, Trieste, Italy; University of Hong Kong, HONG KONG

## Abstract

Dengue virus (DENV), the causative agent of dengue disease, is among the most important mosquito-borne pathogens worldwide. DENV is composed of four closely related serotypes and belongs to the Flaviviridae family alongside other important arthropod-borne viral pathogens such as Zika virus (ZIKV), West Nile virus (WNV) and Yellow Fever virus (YFV). After infection, the antibody response is mostly directed to the viral E glycoprotein which is composed of three structural domains named DI, DII and DIII that share variable degrees of homology among different viruses. Recent evidence supports a close serological interaction between ZIKV and DENV. The possibility of worse clinical outcomes as a consequence of antibody-dependent enhancement of infection (ADE) due to cross-reactive antibodies with poor neutralisation activity is a matter of concern. We tested polyclonal sera from groups of female Balb/C mice vaccinated with DNA constructs expressing DI/DII, DIII or the whole sE from different DENV serotypes and compared their activity in terms of cross-reactivity, neutralisation of virus infection and ADE. Our results indicate that the polyclonal antibody responses against the whole sE protein are highly cross-reactive with strong ADE and poor neutralisation activities due to DI/DII immunodominance. Conversely, anti-DIII polyclonal antibodies are type-specific, with no ADE towards ZIKV, WNV and YFV, and strong neutralisation activity restricted only to DENV.

## Introduction

Dengue virus (DENV) is one of the most important human viral pathogens worldwide [1]. Dengue infections are a major public health concern with significant socioeconomic consequences, particularly in developing countries. The disease is endemic in tropical regions around the world, with more than 3.9 billion people at primary risk of infection; current estimates indicate that around 390 million people have DENV infections each year which result in more than 96 million symptomatic cases [2].

DENV is composed of 4 closely related serotypes (DENV1-4) and belongs to the Flaviviridae family that includes other important and closely related arthropod-borne human viral pathogens such as Zika virus (ZIKV), West Nile virus (WNV), Yellow Fever virus (YFV), Japanese Encephalitis virus (JEV) and Tick-Borne Encephalitis virus (TBEV). Like all flaviviruses, DENV is an enveloped virus with a single-stranded, positive sense RNA genome of ≈11 Kb encoding a single viral polyprotein which renders 10 mature viral proteins: 3 structural (Capsid (C), pre-membrane (PrM) and envelope glycoprotein (E)) and 7 non-structural (NS) proteins (NS1,-2A,-2B,-3,-4A,-4B and -5) [3]. E protein is the principal constituent of the viral particle, playing key roles during viral assembly and endosomal-mediated virus internalisation [4]. The E ectodomain, also termed soluble E (sE), comprises approximately the first 400 aminoacids and is formed by three different structural domains named DI, DII and DIII. DI is a β-barrel structure located at the centre of the E monomer while DII forms an elongated finger-like structure that carries the internal fusion loop. DIII is an Ig-like domain and has been implicated in binding to cellular receptors [5, 6].

E protein is also the main antigenic target of the human antibody response [7]. Neutralising epitopes have been described on all three domains, with domain-specific antibodies displaying different degrees of cross-reactivity among DENV serotypes and other flaviviruses [8–10]. Antibodies against DIII are generally described to be highly neutralising; coincidently, this region shows also the highest variability between serotypes inducing antibodies that are usually highly specific [8, 9, 11, 12]. Recently, new types of potent cross-neutralising antibodies against complex quaternary epitopes, conserved among different viruses and restricted to the dimeric conformation of E or the surface of the whole viral particle have been described and are thought to be the main drivers of viral neutralisation after infection in humans [13–17]. On the other hand, antibodies against epitopes on DI and DII (DI/DII) are, generally, poorly neutralising and highly cross-reactive with other flaviviruses [18]. Interestingly, antibody responses from flavivirus-infected individuals are dominated by highly cross-reactive antibodies mainly focused to antigenic determinants around DII, especially the fusion loop, with relatively reduced contribution of antibodies against DIII or the more complex quaternary epitopes [19–21].

Development of an efficient vaccine against DENV has been a high priority. CYD-TVD, a tetravalent live YF17D-based chimeric vaccine, is the first licensed candidate available worldwide. Its effectiveness, however, has been questioned due to the lack of homogenous protection against all 4 DENV serotypes and a signal of increased risk of severe dengue manifestations in young children [22–24]. In dengue disease, one of the main challenges is the need to develop a single vaccine formulation that confers a balanced, long-lasting and highly neutralising response against all 4 serotypes. This is mainly due to the risk of enhancing infection through a phenomenon known as antibody-dependent enhancement of infection (ADE). In ADE, recognition of viral particles by cross-reacting antibodies with non-neutralising activity or at sub-neutralising concentrations leads to an increased Fcγ receptor-mediated uptake of opsonized particles by monocytes, macrophages and dendritic cells, which results in an increased and more severe infection [25].

Until recently, ZIKV infections were sporadic, mostly asymptomatic and restricted to small geographical areas. However, the virus has rapidly spread to a global scale similar to that of DENV, and is now associated with severe neurological and developmental conditions in humans [26, 27]. DENV and ZIKV are closely related: while the aminoacid sequence of E among DENV serotypes differs by 30–35%, DENV and ZIKV E sequences differs by 41–46%, which explains the significant antigenic overlap between them [7, 28]. Even though previous studies have indicated the possibility of ADE among heterologous flaviviruses [29, 30], it was only recently shown that poorly-neutralising cross-reactive antibodies against DENV are able to enhance ZIKV infection and *vice versa* [31, 32]. In contrast to previous reports based on the study of monoclonal antibodies [32, 33], here we show the activities of polyclonal sera against DENV E protein obtained by domain-based DNA-immunisations in mice. Immunocompetent mouse models, like Balb/c, have been historically used to study the antibody response against DENV vaccines [34, 35]. We used this model to describe how the immune response towards different E domains contributes to virus enhancement and neutralisation of DENV, ZIKV, WNV and YFV, thus providing valuable information for proper antigen design.

## Materials and methods

### Cell lines and viruses

Vero cells (ATCC CCL-81, Rockville, MD, USA) HEK293 cells (ATCC, CRL-1573) and HEK293T/17 cells (HEK293-T, ATCC CRL-11268) were cultured in Dulbecco's modified Eagle's medium (DMEM, Life Technologies, Paisley, UK) supplemented with 10% heat-inactivated foetal calf serum (FCS) (Life Technologies), 50 μg/ml gentamycin and 2 mM L-glutamine. K562 cells (ATCC CCL-243) were maintained in RPMI 1640 medium (Life Technologies) supplemented with 10% FCS and 50 μg/ml gentamycin. Cell cultures were grown at 37°C with 5% of CO_2_.

ZIKV 976 Uganda strain, WNV New York 1999 strain and YFV French neurotropic virus strain (provided by Alessandro Marcello, ICGEB, Trieste, Italy) and DENV1 Hawaii A strain, DENV2 NGC strain, DENV3 isolate 3140/09 and DENV4 TC25 strain, were used. All viral strains were propagated in Vero cells in DMEM containing 2% FCS.

### Plasmid DNA constructs

DENV E glycoprotein sequences were obtained from DENV1 Nauru Island strain (GenBank accession number U88535.1), DENV2 New Guinea C strain (AF038403), DENV3 3H87 strain (M93130), and DENV4 Dominica strain (AF326573.1). Codon-optimised DIII sequences of all DENV serotypes (E protein codons 297–416 for DENV1, DENV2 and DENV4; and 295–414 for DENV3), and sE from DENV3 (codons 1–414) and DENV4 (codons 1–416) were obtained as synthetic fragments (GenScript, Piscataway, NJ, USA). Each DIII and sE sequence was fused to an amino-terminal immunoglobulin leader sequence (sec) [36] and to a carboxy-terminal SV5 tag (GKPIPNPLLGLD) [37]. DIII-SV5 constructs also contained the human IgG heavy chain constant domain 3 (γCH3) downstream of the SV5 tag [38]. The DI/DII-SV5 constructs (codons 1–294 for DENV3 and 1–296 for DENV4) were obtained by removing the DIII coding regions from the sE-SV5 plasmids of DENV3 and DENV4 [39]. All constructs were cloned in pVAX (Thermo Fisher Scientific, Waltham, MA, USA) vector. To produce mono-biotinylated DIII-εCH4 recombinant proteins for ELISA, DIII segments were cloned upstream of the human IgE heavy chain constant domain 4 (εCH4), followed by the biotin acceptor peptide (BAP) sequence (GLNDIFEAQKIEWHE) [40, 41], into a bigenic vector containing the gene for a secretory *E*. *coli* biotin ligase [41]. Likewise, mono-biotinylated versions of DENV3 and DENV4 sE and DI/DII were fused upstream of the BAP tag into the same bigenic vector.

### Animal immunisations

5–6 weeks old, female, Balb/c mice were purchased from Harlan (Milan, Italy) and kept in an isolated hygienic facility, housed in 8 groups of 8 animals within IVC cages (Allentown, Tecniplast Sealsafe Blue series, 1500 cm^2^) under a 12 h light/dark cycle at 22±5°C, with unrestricted access to bedding (Tapvei, 4HP, Paekna, Estonia), food (Envigo, 2018S, Madison, WI, USA) and water, and monitored three times a week throughout the course of the study. Animals were immunised three times at two weeks intervals (Days 1, 15 and 30) by biolistic delivery of 1 μm gold particles coated with 1 μg of plasmid DNA using Gene Gun technology (Bio-Rad, Hercules, CA, USA); in the case of the DIII-tetravalent formulation, animals were vaccinated with two-1 μg DNA shots as previously described [38]. No signs of illness or distress were found following immunisation. Blood samples were collected at day 90 by sub-mandibular puncture and mice were finally euthanized following the CO_2_ inhalation method. De-complemented pooled sera samples (30 min. at 56^°^C) were stored at -20°C until use.

### Expression of recombinant dengue proteins

Transfections of HEK293T/17 cells were performed with standard calcium phosphate method as previously described [42]. Cellular extracts were prepared in 100 μl of TNN lysis buffer (100 mM Tris-HCl, pH 8, 250 mM NaCl, 0.5% NP-40) at 4°C, supplemented with Protease Inhibitor Cocktail (Sigma-Aldrich, St. Louis, MO, USA).

### Western blot

Samples were separated by 10% SDS-PAGE and then transferred to polyvinylidene difluoride (PVDF) membranes (Millipore, Temecula, CA, USA). After blocking with 5% Milk in PBS, membranes were incubated for 1h with anti-SV5 mAb (1:10,000 dilution) [37], washed, and probed for 1h with HRP-linked anti-mouse IgG goat antibodies, (KPL, Gaithersburg, MA, USA, 074–1809). Mouse HRP-conjugated anti-actin mAb (clone AC-15, Sigma-Aldrich) was used as loading control. Signals were developed by ECL (ThermoFisher-Pierce, Rockford, IL, USA).

### ELISA

Recombinant mono-biotinylated proteins were obtained from dialyzed supernatants of stably transfected HEK293 cells clones as previously reported [38]. The relative concentrations of biotinylated proteins were normalized by western blot and ELISA, and comparable amounts of mono-biotinylated DIII-εCH4-BAP, DI/DII-BAP and sE-BAP proteins were captured on Nunc Maxi Sorp Immuno-Plates (ThermoFisher-Nunc, Roskilde, Denmark) previously coated with 100 μl/well of 5 μg/ml avidin (Sigma-Aldrich) in 50mM Na_2_CO_3_/NaHCO_3_ buffer. ELISA assays were performed using different dilutions of sera from immunised mice and revealed using HRP-linked anti-mouse IgG γ-chain goat antibodies (Jackson ImmunoResearch, Newmarket, UK, 115-035-071) and TMB substrate (Sigma), as previously described [38]. Antigen-specific IgG titres are expressed as the reciprocal dilution at which the optical density of sample at a 450 nm wave length (OD_450_), was 3 times higher than that obtained for the pre-immune control serum. Pre-immune sera showed the same performance as negative control sera from animals DNA-immunised with an irrelevant protein fused to γCH3. Avidity indexes were determined as previously described [38] using a modified ELISA protocol to determine the relative change between OD_450_ values obtained with and without washings in 6M urea, expressed as percentages. In agreement with previous reports, avidity indexes above 30% were considered high [43].

### Immunofluorescence

Vero cells were infected with virus preparations at a MOI of 0.5 for 36h (24h for WNV), fixed with 3.7% paraformaldehyde (PFA) in PBS for 20 min, quenched with 150 mM glycine in PBS, permeabilised with 1% Triton in PBS for 15 min, blocked with 0.1% BSA in PBS for 1h and then incubated with anti-DIII, anti-DI/DII or anti-sE sera (1:100 dilution), mAb 4G2 (Millipore, Temecula, CA, USA; 50 ng/ul) or pre-immune control sera, followed by Alexa488-conjugated goat anti-mouse IgG (Jackson ImmunoResearch, Newmarket, UK, 1:1000). Labelled cells were mounted with ProLong supplemented with DAPI (Thermo Fisher Scientific). Images were acquired using a Nikon Eclipse Ti-E microscope.

### Foci reduction neutralisation test (FRNT)

FRNT was carried out in 48 multi-well plates, on Vero cells seeded at a density of 6.5x10^4^ cells/well 24h before infection. Sera samples were 2-fold serially diluted and incubated for 1.5h at 36^°^C with an equal volume of DMEM serum-free medium containing 50 foci-forming units (FFU) of each virus. Vero cells were then infected in duplicate for 1h at 36°C, the inoculum was then removed and cells overlaid with 0.5 ml of DMEM containing 2% FCS and 3% carboxymethylcellulose (Sigma). After 3 days incubation at 36°C, cell monolayers were washed and fixed for 20min with PFA 3.7%, permeabilised with a 1% Triton solution in PBS for 10 min and treated with 0.3% H_2_O_2_ in methanol for 30 min. Infection foci were developed incubating with mAb 4G2 (1 ng/μl) for 1h at room temperature (RT), followed by incubation with HRP-conjugated goat anti-mouse IgG (Jackson ImmunoResearch, 1:1000). Foci were then stained with 100 μl True-Blue reagent (KPL, Gaithersburg, MA, USA) and counted. Neutralising antibody titres were expressed as the serum dilution yielding a 50% foci reduction when compared against pre-immune sera (FRNT_50_).

### Antibody-dependent viral infection in K562 cells

10-fold dilutions of sera or mAb 4G2 were incubated with an equal volume of RPMI 1640 serum-free medium containing 4x10^3^ FFUs of each virus and incubated for 1.5h at 36^°^C in round-bottom 96 multi-well plates (Corning-Costar, Corning, NY, USA). 4x10^4^ K562 cells (0.1 MOI) were added to the sera-virus mixture and incubated for 72 h (48 h for WNV) at 36^°^C. Cells were then fixed for 30 min with 2% PFA on ice, blocked in permeabilisation buffer (0.1% saponin (Sigma-Aldrich), 2% FBS and 0.1% NaN_3_ in PBS) for 30 min at 4°C and incubated with mAb 4G2 (1 ng/ul in permeabilisation buffer) followed by Alexa 488-conjugated goat anti-mouse IgG (Jackson ImmunoResearch, 1:1000 in permeabilisation buffer), both for 1h at RT. After washing, cells were resuspended in PBS containing 2% FBS and 0.1% NaN_3_ and analysed by cytofluorimetry in a FACSCalibur (BD Biosciences, San Jose, CA, USA).

### Statistical analysis

In all cases, data were obtained from a minimum of three independent experiments (n) done in triplicate. Serological experiments were performed using pooled sera from groups of immunised mice. Unless indicated otherwise, arithmetic means ± standard deviations were calculated and analysed using unpaired two-tailed *t* test when needed (GraphPad Prism 6.0, GraphPad Software Inc., La Jolla, CA, USA); *p* values <0.05 were considered significant and variances between compared groups were not significantly different. Sample size was not statistically assessed and data distribution was assumed to be normal. Data collection or analysis was not blinded.

### Ethical statement

All animal procedures described in this study were approved by the ICGEB Animal Welfare Board and the Italian Ministry of Health (Ministero della Salute) (approved protocol DGSAF0024706) and conducted adhering to institutional and international guidelines for animal experimentation, and in compliance to laws and policies established in the legislation D. L.vo 26/2014 of the Italian Government.

## Results

### Immunological dominance of DENV E protein DI/DII epitopes

We have previously reported antibody responses in mice following DNA immunisations with DENV sE protein (anti-sE) or with only domain III (anti-DIII). DIII of all four DENV serotypes were engineered as a fusion with the human IgG CH3 dimerising domain to allow optimised expression and secretion from mammalian cells [38]. Upon immunisations, these constructs induced anti-DIII responses with strong neutralising activities for the homologous DENV serotype and reduced cross-reactivity and ADE for the non-homologous serotypes [38]. In contrast, anti-sE antibodies from mice immunised with DENV3 sE, showed low anti-DIII activity with most of the antibody response directed against domains I and II (DI/DII) [38], in agreement with previous studies [19, 44].

**Fig 1:**
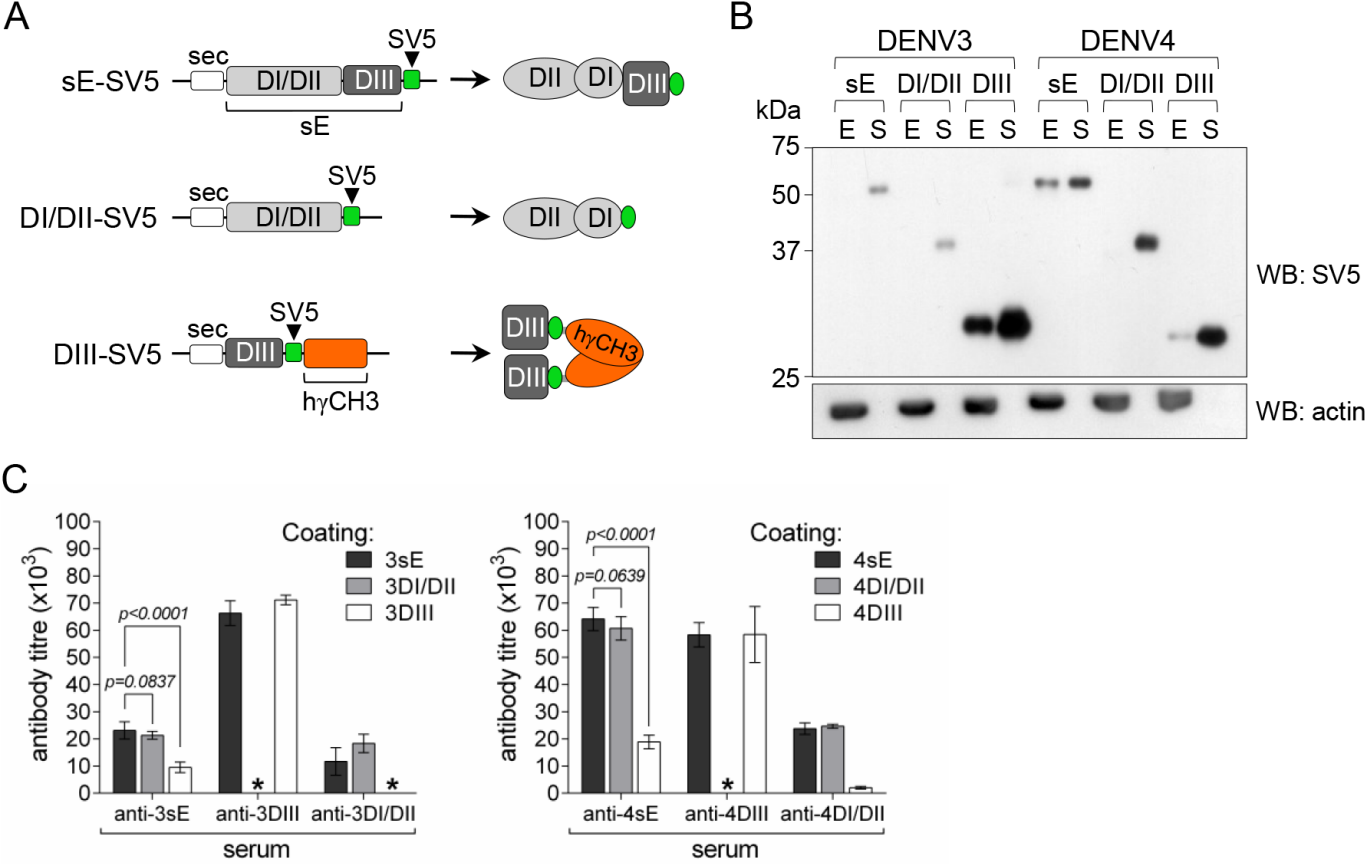
Antibody responses against DENV sE antigens are highly dominated by DI/DII epitopes. A) Scheme of DNA constructs and the expected products used for immunisations. B) Western blot profile of expression and secretion from transfected HEK293T cells (E, cellular extracts; S, culture supernatants) of sE-SV5, DI/DII-SV5 and DIII-SV5 constructs from DENV3 and DENV4; anti-actin was used as loading control. C) ELISA antibody titres of pooled sera from mice immunised with the indicated constructs determined on plates coated with homologous sE, DIII or DI/DII antigens from DENV3 (left panel) and DENV4 (right panel) as indicated (in each case, n = 4. Comparisons of anti-sE reactivity against sE and DI/DII antigens (t = 1.172 df = 6 for DENV3, t = 1.541 df = 6 for DENV4), and against sE and DIII antigens (t = 8.160 df = 6 for DENV3, t = 28.22 df = 6 for DENV4) are shown. *, indicates antibody titre below pre-immune control.

To further confirm DI/DII immunodominance, we tested sera from mice immunised with constructs encoding the whole sE protein or only DI/DII or DIII (schematically shown in Fig 1A). While DIII from all 4 DENV serotypes were studied, only sE and DI/DII constructs of DENV3 and DENV4 were included as the encoded proteins were previously shown to be properly folded and efficiently secreted from transfected mammalian cells, contrary to those of DENV1 and DENV2 that are poorly secreted, thus inducing significantly low immune responses upon DNA vaccination [39].

Immune responses against sE, DI/DII and DIII of DENV3 and DENV4 were compared by ELISA on plates coated with normalised amounts of the different homologous antigens. In agreement with previous studies [38, 39], antibody titres obtained from DNA-immunised animals were directly dependent on the relative amount of antigen secreted from transfected mammalian cells (Fig 1B). As shown in Fig 1C, anti-DI/DII and anti-DIII sera showed high specificity against their respective domains while anti-sE antibodies were mostly directed against DI/DII, thus confirming DI/DII immunodominance in relation to DIII. In both cases, there was no significant difference between anti-sE antibody titres determined on plates coated with sE or with DI/DII. Conversely, the difference was highly significant when comparing anti-sE titres on sE- or DIII-coated plates. As expected, antibodies directed against the human γCH3 domain did not cross-react with the εCH4 thus confirming specificity of the assay (S1 Fig). The reactivity curves of each pooled sera against the different antigens are shown in panel A of S2 Fig; in all cases anti-DI/DII responses showed a significantly reduced avidity index (Panel B of S2 Fig).

### Antibodies against DI/DII, but not against DIII are highly cross-reactive

**Fig 2:**
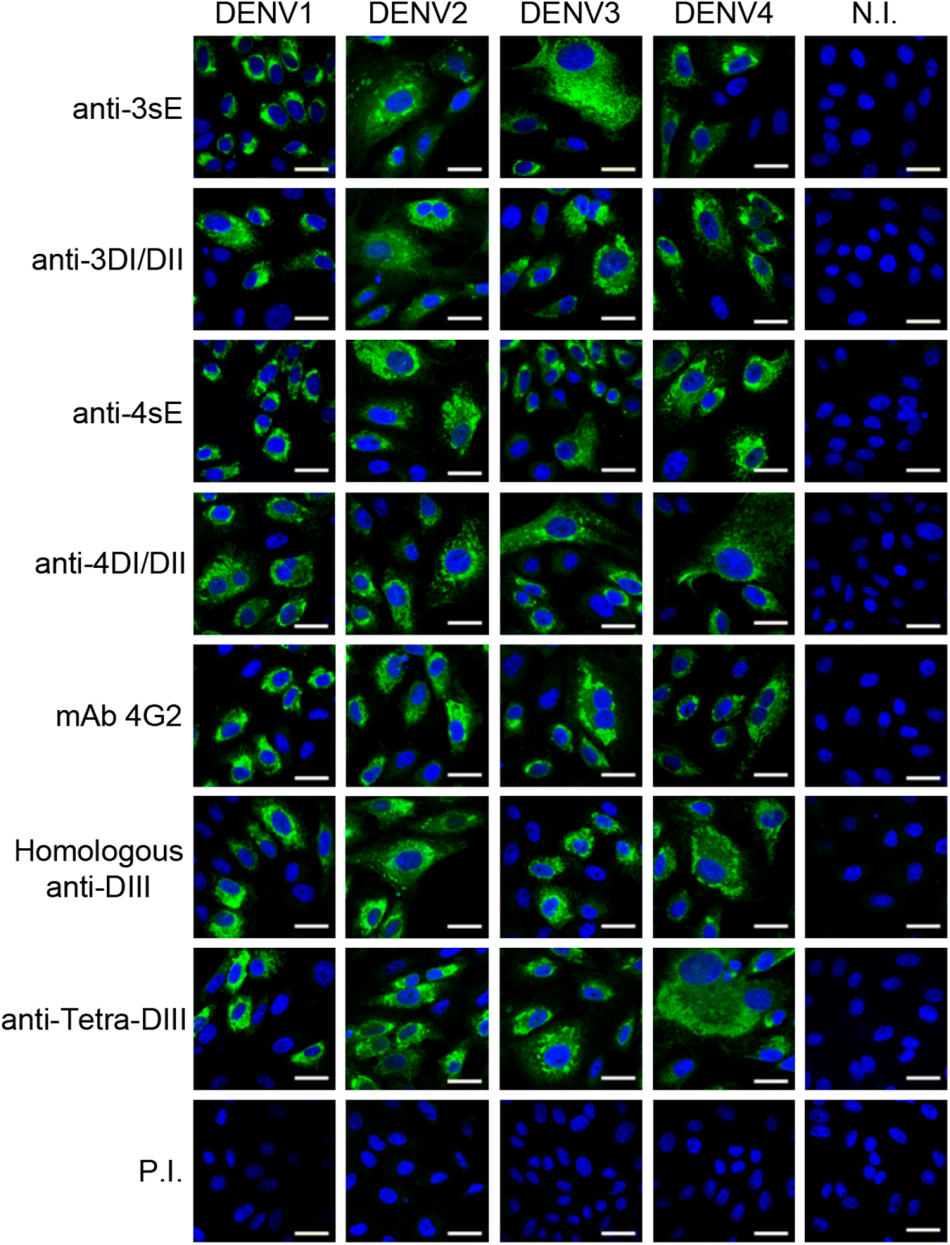
Reactivity profiles of anti-DENV sera in DENV-infected cells. Immunofluorescence of Vero cells infected with all four DENV serotypes and reacted with antibodies elicited with 3sE and 4sE or the corresponding 3DI/DII and 4DI/DII. mAb 4G2, the homologous anti-DIII and the tetravalent anti-DIII sera were included as controls to show reactivity towards each serotype. N.I., non-infected cells; P.I., pre-immune serum. Bar, 30 μm. Representative images from independent experiments are shown.

**Fig 3:**
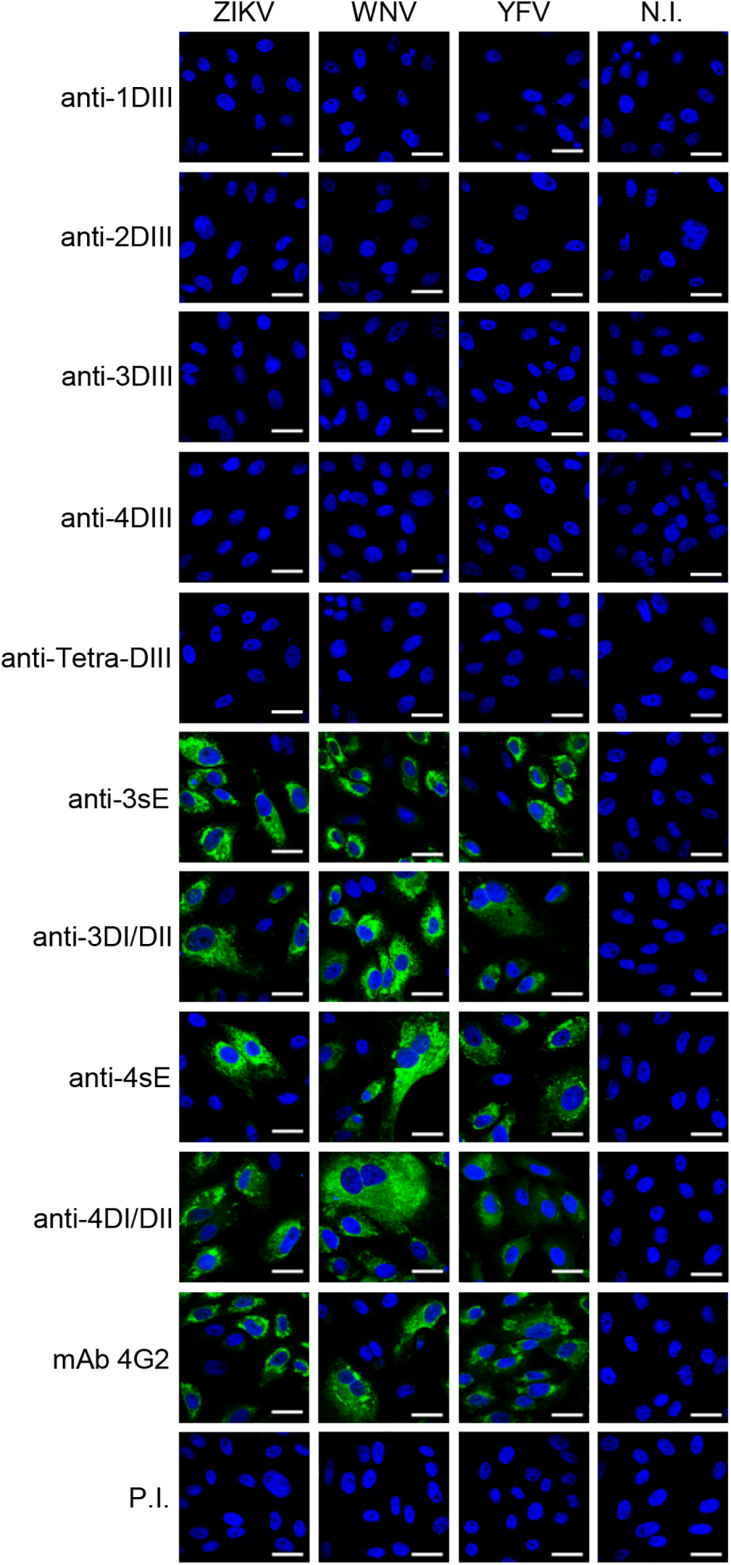
DENV anti-DIII sera do not cross-react with ZIKV, WNV and YFV. Immunofluorescence of Vero cells infected with ZIKV, WNV and YFV reacted with antibodies elicited against each of the four DENV DIII antigens (1DIII, 2DIII, 3DIII and 4DIII), the Tetra-DIII formulation, 3sE, 3DI/IDII, 4sE and 4DI/DII. mAb 4G2 was included as a control. N.I., non-infected; P.I., pre-immune serum. Bar, 30 μm. Representative images from independent experiments are shown.

We have previously reported a detailed analysis on the cross-reactive properties of the different anti-DIII responses against all four DENV viral serotypes [38]. Based on the specificity of anti-DIII responses, we first investigated cross-reactivity of antibodies specifically induced with DIII (of all 4 DENV serotypes), and with DI/DII and sE proteins (of DENV3 and DENV4) by immunofluorescence in cells infected with the four DENV serotypes (Fig 2) and with three other flaviviruses: ZIKV, WNV, and YFV (Fig 3). Reactivity of each anti-DIII sera against its homologous DENV serotype was included to show the presence of reactive antibodies in the monovalent formulation. Since vaccine candidates against DENV are required to elicit a protective response against all 4 serotypes, we also included sera from animals immunised with a tetravalent formulation of the DIII-based vaccine to properly assess the reactivity of the potential candidate (Tetra-DIII) [38].

As expected, monovalent anti-DIII antibodies showed high reactivity towards their homologous serotypes. Likewise, sera from mice immunised with Tetra-DIII clearly recognised all four DENV serotypes (Fig 2). In addition, both anti-sE and anti-DI/DII sera were highly cross-reactive against all DENV serotypes (Fig 2). Interestingly, when tested on cells infected with the three other flaviviruses (ZIKV, WNV and YFV), anti-DIII antibodies (either serotype-specific or Tetra-DIII) were completely negative, whereas antibodies induced with sE or DI/DII were strongly positive in all cases, further supporting not only the immunodominance of DI/DII epitopes, but also the high degree of cross-reactivity of these antibodies (Fig 3). For all viruses, mAb 4G2, which recognizes the fusion loop of all flaviviruses [45], and pre-immune sera were used as positive and negative controls, respectively.

### ADE and neutralisation activities of anti-DI/DII and anti-DIII sera

**Fig 4:**
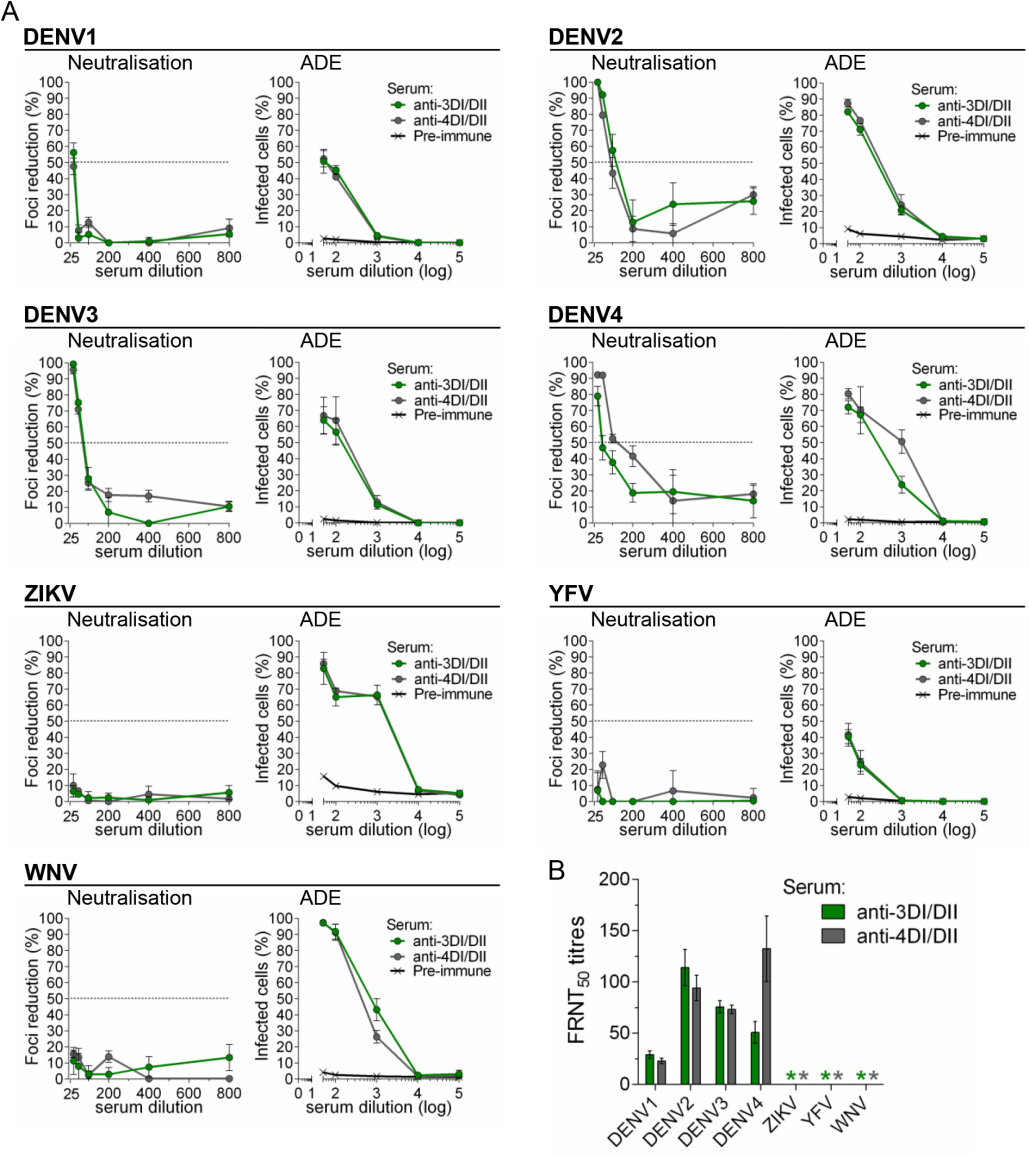
DENV DI/DII induces poorly-neutralising, highly-enhancing cross-reactive antibodies. A) Neutralisation and ADE of all four DENV serotypes, ZIKV, WNV and YFV, with DENV anti-3DI/DII and anti-4DI/DII pooled sera (in all cases, n = 3 for each virus-sera combination). B) FRNT_50_ titres, as determined in (A), * indicates FRNT_50_ titres below detection limit of the assay (≥25).

**Fig 5:**
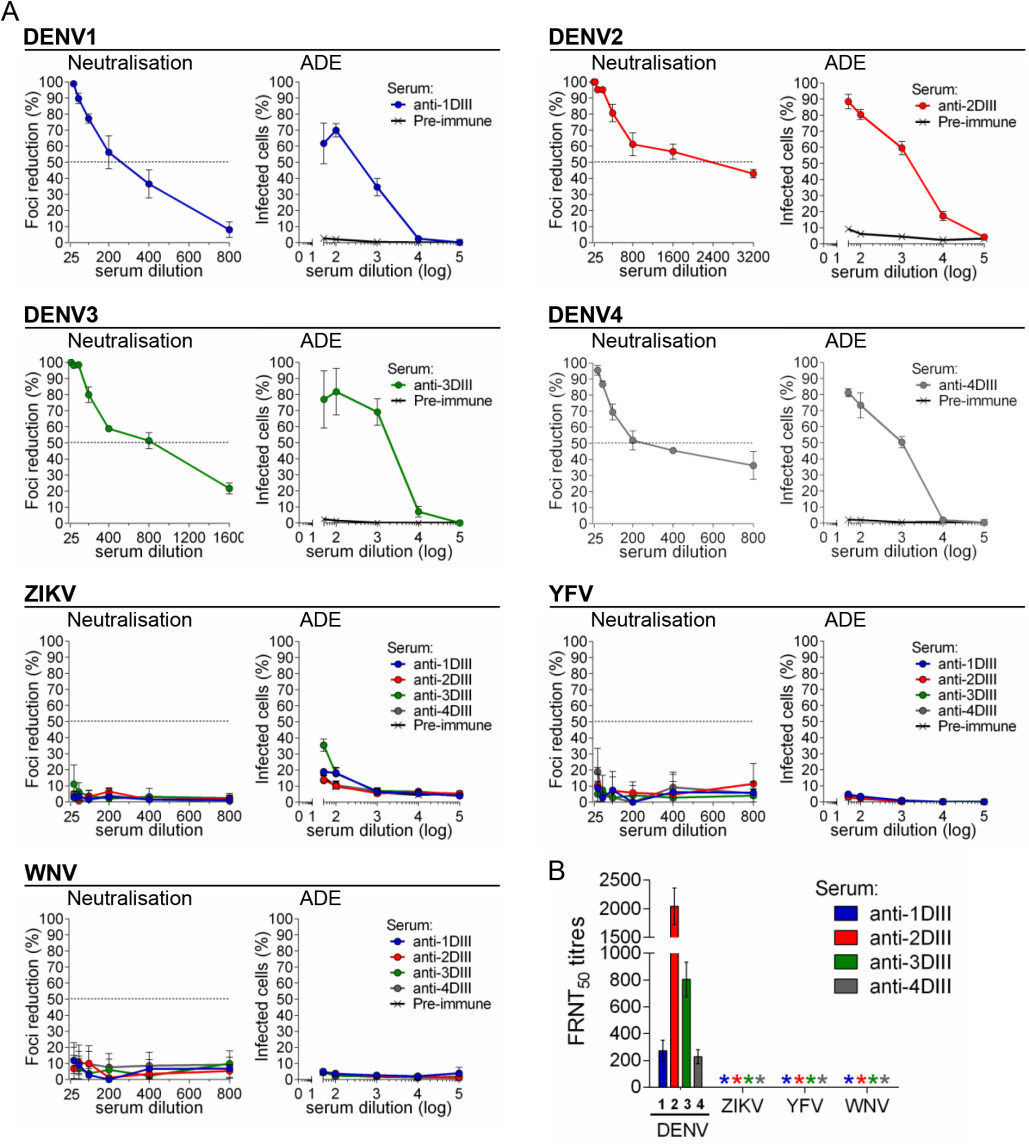
DIII vaccination induces DENV-specific neutralising antibodies. A) Neutralisation and ADE of all four DENV serotypes, ZIKV, WNV and YFV, with DENV anti-DIII sera. To prove anti-DIII reactivity, DENV viruses were tested using pooled sera against the homologous serotypes, while non-DENV viruses were tested against all four anti-DIII sera (in all cases, n = 3 for each virus-sera combination). B) FRNT_50_ titres, as determined in (A), * indicates FRNT_50_ titres below detection limit of the assay (≥25).

In order to dissect the properties of polyclonal antibody responses elicited by the different E structural domains, and because of the broad cross-reactivity of anti-DI/DII (and anti-sE) sera in contrast to the relatively high specificity of anti-DIII, we subsequently tested ADE and neutralisation activities of the different sera in K562 and Vero cells, respectively. Since anti-DI/DII and anti-sE responses (for both DENV3 and DENV4) showed the same reactivity profiles, we only compared anti-DI/DII with anti-DIII specific sera. Antibodies elicited against DI/DII of DENV3 and DENV4 (anti-3DI/DII and anti-4DI/DII, respectively) showed strong ADE on all four DENV serotypes as well as on ZIKV, WNV and YFV (Fig 4A). Neutralisation activity, however, was only relevant for DENV serotypes and negative on all other viruses (Fig 4A and 4B). In contrast, anti-DIII antibodies showed no ADE (Fig 5A) and no neutralisation (Fig 5A and 5B) on ZIKV, WNV and YFV. As expected, each anti-DIII serum was able to neutralise and induce ADE on the homologous DENV serotype. As a control of the ADE assay, all tested flaviviruses showed ADE when incubated with mAb 4G2 (S3 Fig).

**Fig 6:**
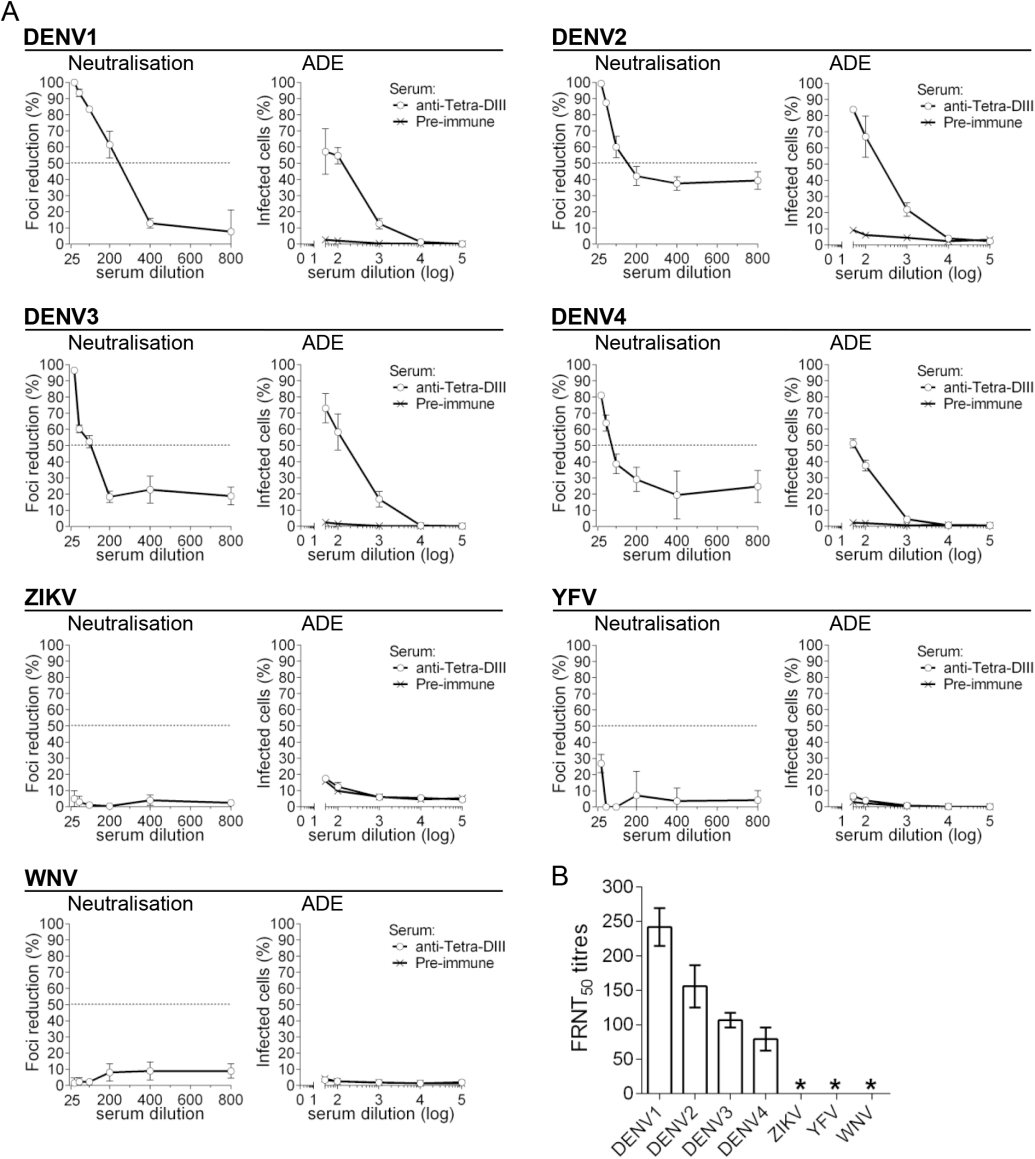
DENV-DIII tetravalent vaccine formulation elicits DENV-specific neutralising antibodies. A) Neutralisation and ADE of all four DENV serotypes, ZIKV, WNV and YFV, with pooled sera from mice vaccinated with the Tetra-DIII formulation (in all cases, for each virus-sera combination n = 3). B) FRNT_50_, as determined in (A), * indicates FRNT_50_ titres below detection limit of the assay (≥25).

We then determined ADE and neutralisation activities of sera from mice vaccinated with Tetra-DIII. Not surprisingly, Tetra-DIII sera showed neutralisation activity for all four DENV serotypes but not for the other flaviviruses (Fig 6A). More importantly, ADE was completely negative on the non-DENV flaviviruses, and positive on the four DENV serotypes, as expected (Fig 6A). Neutralisation titres are shown in Fig 6B.

Thus, our data strongly support the specificity of anti-DIII polyclonal responses, and further highlight the importance of inducing anti-DIII antibodies as a source of effective DENV-specific neutralisation activity without promoting ADE of other closely related flaviviruses such as ZIKV, WNV and YFV.

## Discussion

Patients with a secondary DENV infection are at a higher risk of suffering the more severe haemorrhagic manifestations of the disease. In fact, severe symptoms following DENV infections are 15–80 times more frequent in secondary infections, and pre-existing heterotypic DENV antibodies are found in almost all patients with severe disease [25]. ADE was initially proposed to explain this phenomenon and concern over its role in DHF/DSS has justified the requirement for all DENV vaccines candidates to induce a balanced and long-lasting protective response against all serotypes at the same time. Given that the current ZIKV outbreak overlaps many DENV-endemic areas and the high degree of homology between DENV and ZIKV, there is great concern on how the outcome of ZIKV infections might be altered in DENV immune patients [46, 47]. Recent studies have demonstrated that pre-existing anti-DENV antibodies enhance ZIKV infection *in vitro* and *in vivo*, suggesting that DENV immunity could represent a risk to develop more severe symptoms upon ZIKV infection [31, 48]. The complex serological relationship between DENV and ZIKV was further confirmed by showing reciprocal *in vitro* and *in vivo* ADE following isolation of monoclonal antibodies from infected patients [28, 32]. Moreover, due to significant antigen overlapping, this could also apply to infections by other flaviviruses like WNV and YFV [30, 48].

Together, these results indicate a complex serological interaction between flaviviruses, with serious implications regarding the design and evaluation of future DENV and ZIKV vaccines. Most notably, these findings introduce the need to consider the relative activity of antibodies in terms of ADE among closely related flaviviruses, particularly when cross-reactive antibodies are present.

We have previously reported an efficient way to induce strong antibody responses against all four DENV serotypes, based on a DNA-vaccine with DIII, properly engineered to significantly increase expression and secretion in mammalian cells as immunising antigen [38]. The antibody responses showed high neutralisation titres and reduced ADE among the different DENV serotypes in THP-1 cells that express high levels of FcγRI, FcγRIIA and FcγRIIB [49].

Here, instead, we used K562 cells, which are FcγRI negative and express high levels of FcγRIIA that significantly favours FcγR-mediated infection, thus allowing us to evaluate the ADE potential of the different sera with higher sensitivity as these cells are particularly prone for ADE-mediated infection [49]. In this context, sera unable to induce ADE in K562 cells are highly unlikely to do so in other less susceptible cells. Our new data indicate that only anti-DIII antibodies show high type-specificity as they did not recognize other non-DENV flaviviruses. As a consequence, DENV anti-DIII antibodies, even in the context of a tetravalent vaccine formulation, do not promote ADE of WNV, ZIKV and YFV. In contrast, anti-DI/DII antibodies are highly cross-reactive with strong ADE activity for WNV, ZIKV and YFV and without significant neutralisation activity. This is an important point to consider as ADE is always present, even with antibodies with strong neutralising activity particularly at non-neutralising concentrations, as a consequence of the natural waning of antibody levels in time after infection or vaccination [50].

The development of a vaccine efficiently preventing DENV infection while avoiding ADE of the different serotypes and other closely related flaviviruses remains an unmet challenge. Our data indicate that DIII-based vaccines could avoid the risk of inducing ADE of other closely related flaviviruses. They also stress the DI/DII immunodominance with respect to DIII when using the whole sE as immunising antigen, resulting in poorly-neutralising, highly cross-reactive and ADE promoting antibodies. In addition, anti-DI/DII responses showed significantly low avidity, a feature that has been shown to negatively correlate with the neutralising capacity of antibodies [51, 52]. These results are in agreement with published data indicating that cross-reactive antibodies against the virion are the main drivers of heterotypic DENV enhancement of infection [13, 44]. Recently, highly cross-reactive monoclonal antibodies against DI/DII from DENV- and ZIKV-infected patients were shown to be poorly neutralising but potently infection-enhancing [32]. Furthermore, a recent study with monoclonal antibodies obtained from ZIKV-immunised mice, showed that type-specific protective and neutralising antibodies bound mainly to DIII, identifying ZIKV DIII as a potentially safe immunogen for vaccines [33].

Preliminary data on new vaccine candidates against ZIKV rely mostly on a variety of genetic immunisation approaches based on the expression of the E protein together with PrM [53–58]. In some of these cases, however, ADE activities of the induced antibodies against other flaviviruses were not assessed [53–56, 58]. Furthermore, attempts of reducing the risk of ADE in mRNA-based ZIKV vaccine, diminished the efficacy of the vaccine when compared to the original construct, highlighting the complexity involved in proper antigen design [57]. Finally, the introduction of PrM in some of these formulations is a matter of concern, as anti-Pr antibodies have been shown to be also highly cross-reactive with strong ADE activity and almost no neutralising capacity [59].

Despite several studies on responses against DENV and ZIKV after infection, deep understanding of the natural polyclonal antibody response following immunisation with vaccine candidates is lacking. Our study describes the first domain-based characterization of the polyclonal immune response induced against DENV sE protein, by comparing cross-reactivity, neutralisation and ADE activities of antibodies elicited by DNA immunization with constructs expressing DIII or DI/DII separately. By analyzing polyclonal responses, our results confirm previously reported data on DENV and ZIKV using monoclonal antibodies and further highlight DIII as a strong candidate to develop DENV-specific vaccines without the concerns regarding cross-reactivity to other flaviviruses, particularly ZIKV.

As previously reported [38], our tetravalent DIII-based DNA vaccine formulation elicited neutralizing activity against all four DENV virus serotypes. However, in contrast to other DIII-based vaccines [60, 61], there was interference between the different antigens as the FRNT_50_ titres were reduced when compared against the monovalent formulations, thus highlighting the need for further development of the Tetra-DIII formulation in order to achieve balanced, long-lasting and strong neutralising responses.

The description of strong neutralising antibodies against conserved dimer-dependent epitopes has opened the opportunity for the development of a universal vaccine for DENV and ZIKV [17, 28]. However, our data raise questions as to their potential use due to the large involvement of other immunodominant DI/DII epitopes. In this scenario, effective modulation of antigenicity to favour responses against cross-neutralising epitopes would be required. The recent descriptions of covalently-stabilised E dimers from DENV and ZIKV represent an important step towards achieving this goal [62, 63].

Although the true impact of ADE in DENV infection has not been fully elucidated and no animal model has been able to predict the full repertoire of the antibody response in humans [64], this phenomena introduces significant concerns for the development of efficient vaccine candidates. There is abundant evidence supporting the effect of antibody-mediated enhancement and disease between DENV serotypes; however, there are no reports confirming a correlation between *in vitro* ADE assays and increased risk of infection by other flaviviruses; moreover, there is no clinical evidence of increased disease severity from WNV, ZIKV and YFV in DENV-immune patients or *vice versa*. Nonetheless, the risk for haemorrhagic complications found in (CYD-TVD)-vaccinated children, suggests that these concerns are relevant and should be taken under consideration during preclinical and clinical evaluation of potential vaccines. In addition, recent data indicate that host-related factors are also involved in severe dengue associated to ADE [65].

Based on these complex serological interactions, the choice of antigen for the design of vaccine candidates is of paramount importance, as it should be able to induce highly neutralising antibodies with negative or much reduced ADE of other related viruses. These requirements are fulfilled by DENV DIII in the format we have described. Moreover, DNA-vaccine platforms, and genetic vaccines in general, have an excellent safety profile, are highly stable and can be manufactured in a short period of time [66]. Our data support further development of DIII-based DNA-vaccines to induce type-specific neutralising antibodies that circumvent the risk of ADE among flaviviruses.

## Supporting information

S1 Fig ELISA specificity: Lack of cross-reactivity of anti-3DIII-gCH3 sera on eCH4. Sera from animals immunised with 3DIII-CH3, or with an irrelevant protein fused to gCH3 (Irr-gCH3) tested on plates coated with mono-biotinylated 3DIII-eCH4 (left panel), human gCH2-CH3 (centre panel) and Irr-eCH4 (right panel). PI: pre-immune sera.
(TIF)

S2 Fig Antibody responses against DENV sE antigens. A) ELISA profiles of antibody responses from mice immunised with DIII, DI/DII and sE form DENV3 and DENV4, tested on plates coated with the homologous sE, DIII or DI/DII antigens. DENV3, top panels; DENV4, bottom panels (in all cases, n = 12). B) Avidity index of sera from animals immunised with DIII, DI/DII and sE constructs from DENV3 (left) and DENV4 (right) (in all cases n = 18), mean±s.d. are shown.
(TIF)

S3 Fig ADE induced by mAb 4G2 on DENV, ZIKV, WNV and YFV. ADE of all four DENV serotypes, ZIKV, WNV and YFV on K562 cells, using the control mAb 4G2 (in all cases n = 3).
(TIF)

S1 File The NC3Rs ARRIVE guidelines checklist. (PDF)
