# Supplementary figures and images for: DNA-immunisation with dengue virus E protein domains I/II, but not domain III, enhances Zika, West Nile and Yellow Fever virus infection
Source: PLoS One. 2017 Jul 25;12(7):e0181734. doi: 10.1371/journal.pone.0181734 (PMC5526558; doi:10.1371/journal.pone.0181734)

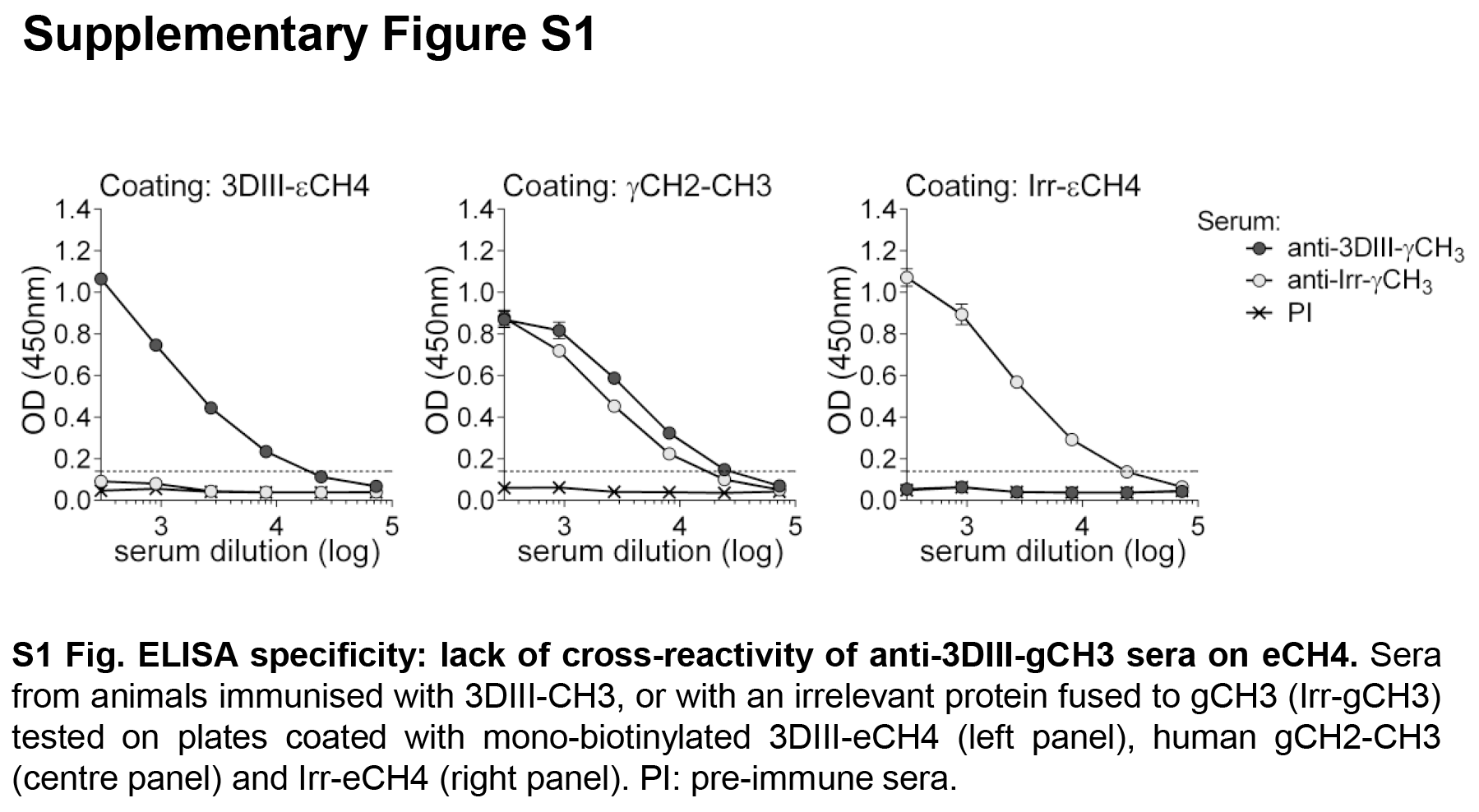

Supplement: S1 Fig — Sera from animals immunised with 3DIII-CH3, or with an irrelevant protein fused to gCH3 (Irr-gCH3) tested on plates coated with mono-biotinylated 3DIII-eCH4 (left panel), human gCH2-CH3 (centre panel) and Irr-eCH4 (right panel). PI: pre-immune sera. (TIF) [file pone.0181734.s001.tif]

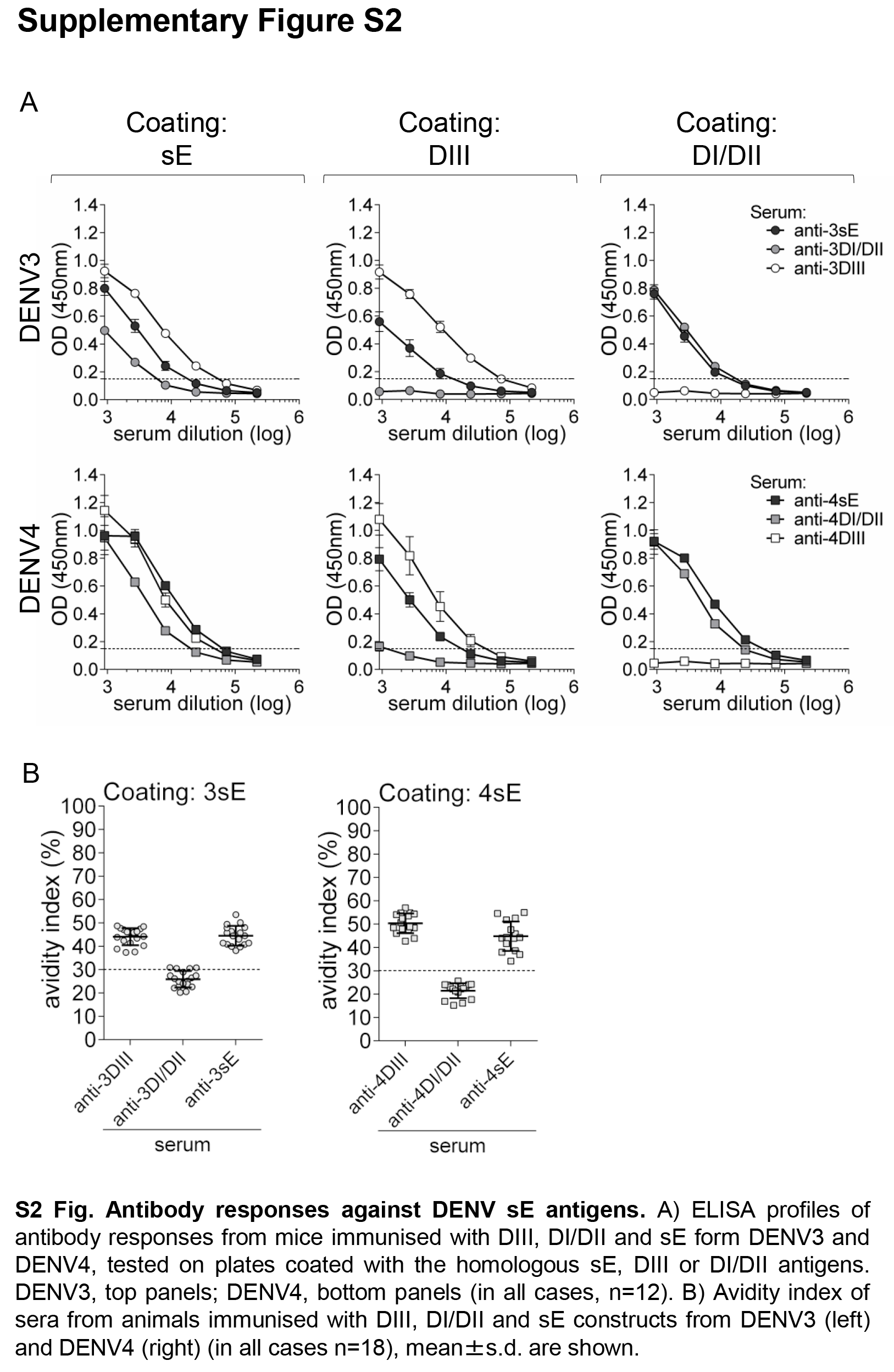

Supplement: S2 Fig — A) ELISA profiles of antibody responses from mice immunised with DIII, DI/DII and sE form DENV3 and DENV4, tested on plates coated with the homologous sE, DIII or DI/DII antigens. DENV3, top panels; DENV4, bottom panels (in all cases, n = 12). B) Avidity index of sera from animals immunised with DIII, DI/DII and sE constructs from DENV3 (left) and DENV4 (right) (in all cases n = 18), mean±s.d. are shown. (TIF) [file pone.0181734.s002.tif]

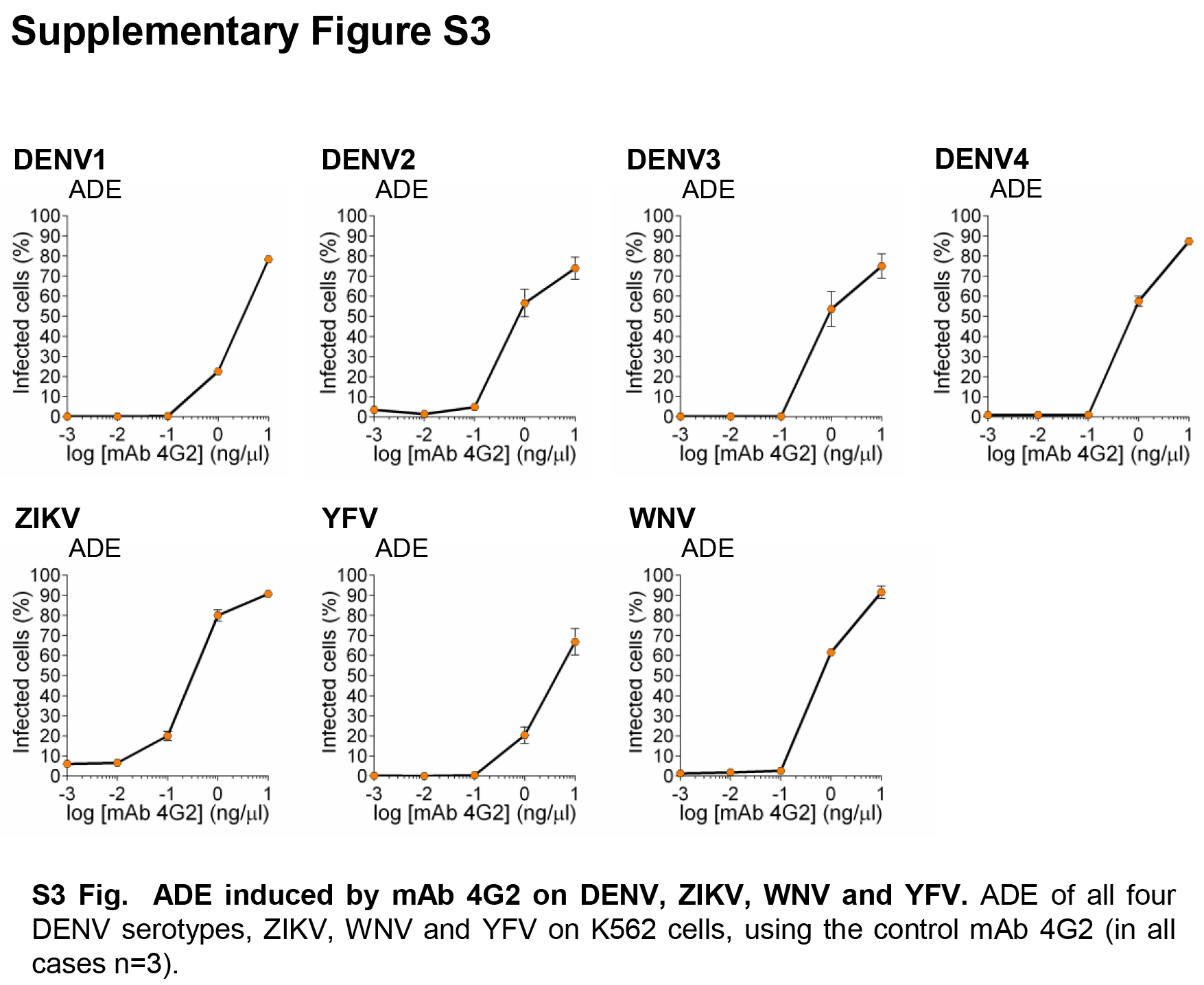

Supplement: S3 Fig — ADE of all four DENV serotypes, ZIKV, WNV and YFV on K562 cells, using the control mAb 4G2 (in all cases n = 3). (TIF) [file pone.0181734.s003.tif]
